# Total Iridoid Glycosides from *Swertia mussotii* Franch. Alleviate Cholestasis Induced by α-Naphthyl Isothiocyanate through Activating the Farnesoid X Receptor and Inhibiting Oxidative Stress

**DOI:** 10.3390/ijms251910607

**Published:** 2024-10-02

**Authors:** Qi Dong, Zhenhua Wang, Na Hu, Fangfang Tie, Zenggen Liu, Ying Sun, Yue Wang, Nixia Tan, Honglun Wang

**Affiliations:** 1 Qinghai Provincial Key Laboratory of Tibetan Medicine Research and CAS Key Laboratory of Tibetan Medicine Research, Northwest Institute of Plateau Biology, Xining 810008, China; qdong@nwipb.cas.cn (Q.D.); huna@nwipb.cas.cn (N.H.); fftie@nwipb.cas.cn (F.T.); lzg2005sk@126.com (Z.L.); 2 College of Life Sciences, Yantai University, Yantai 264005, China; skywzh@ytu.edu.cn (Z.W.); sunny030726@163.com (Y.S.); 3 Medical College, Qinghai University, Xining 810016, China; ys211055001369@qhu.edu.cn (Y.W.); ys221055001405@qhu.edu.cn (N.T.)

**Keywords:** *Swertia mussotii* Franch., iridoid glycosides, cholestasis, FXR, oxidative stress

## Abstract

Cholestasis refers to a physiological and pathological process caused by bile acid (BA) overaccumulation inside the circulatory system and liver, leading to systemic and hepatocellular damage. Activating the farnesol X receptor (FXR) to restore BA homeostasis is a promising strategy for treating cholestasis. The objective of this research is to reveal solid evidence for the fact that the total iridoid glycosides from *Swertia mussotii* Franch. (IGSM) alleviate cholestasis. In this research, the whole plant of *S. mussotii* was extracted with 70% ethanol and separated by macroporous adsorption resin. A rat cholestasis model was established by the injection of α-naphthyl isothiocyanate (ANIT) at a dose of 75 mg/kg. Biochemical and oxidative stress indicators were determined using commercial assay kits. The mRNA abundance of FXR and target proteins was assessed using RT-qPCR. In addition, the effects of main compounds with FXR were evaluated by molecular docking after IGSM analysis using UPLC. The results indicated that IGSM alleviated ANIT-induced cholestasis through reducing serum ALT, AST, AKP, and TBA levels; increasing the mRNA levels of *Fxr*, *Besp*, *Ntcp*, and *Mep2*; and reducing oxidative stress. The proportion of iridoid compounds in IGSM exceeded 50%, which may be the active substance basis of IGSM. This study provides a theoretical reference for IGSM in the treatment of cholestasis, and future studies may delve more deeply into the FXR regulatory pathway.

## 1. Introduction

Cholestasis refers to a physiological and pathological process caused by bile composition (namely bile acid [BA], bilirubin, cholesterol, etc.) overaccumulation inside the systemic circulatory system and liver, leading to systemic and hepatocellular damage. Persistent cholestasis results in fibrosis and even cirrhosis of the liver [1]. The farnesoid X receptor (FXR) is a BA-activated transcription factor that has a pivotal role in BA homeostasis [2,3]. FXR modulates the uptake and synthesis of BA by regulating the levels of proteins involved in the FXR signaling pathway [4]. Under a cholestatic state, FXR increases the solubility of BA and its elimination via facilitating the expression of bile salt export pump (BSEP) and conjugation reactions [5]. Therefore, the drugs targeting FXR to ameliorate liver injury in cholestasis have been extensively studied [5]. Besides that, drug treatments such as PPAR agonists, bile acid inhibitors, anti-fibrotic drugs, and anti-inflammatory drugs are useful in improving the condition of the disease [6]. Ursodeoxycholic acid (UDCA), adrenocortical hormones, and obeticholic acid are the currently used drugs for cholestasis. However, their efficacy and safety are unsatisfactory [7]. Therefore, more effective and safer drugs for cholestasis should be developed. Traditional Chinese medicine has a long history of successful applications in cholestasis treatment and thus is promising.

Zangyinchen, which is also known as Sangti, is the main traditional Tibetan medicine prescription for treating liver diseases. It was recorded in the “Four Classics of Traditional Chinese Medicine”, which was written in the 8th century A.D. The main component in Zangyinchen is *Swertia mussotii* Franch. (Gentianaceae). *S. mussotii* is mainly distributed in Qinghai, Tibet, Yunnan, and western Sichuan, and it is traditionally used to disperse stagnated liver qi for promoting bile flow, promote gallbladder function, eliminate excess heat, and alleviate jaundice (especially jaundice associated with icteric hepatitis and viral hepatitis) [8].

It has been demonstrated that *S. mussotii* significantly reduces the levels of liver injury indicators such as aspartate transaminase (AST) and alanine transaminase (ALT) in mouse serum and alleviates acute liver injury by modulating lipid metabolism-associated pathways and improving the microcirculation in the mesentery and liver [8,9]. Moreover, *S. mussotii* inhibits liver fibrosis and alleviates CCl4-induced liver injury [10]. *S. mussotii* and its main compound (gentiopicroside) alleviated liver injury triggered by cholestasis in rats, possibly through increasing BA transporter levels, such as those of multidrug resistance-associated protein 2 (MRP2) and BSEP on the canalicular membrane of hepatocytes [11]. The alcohol extract of *S. mussotii* significantly reduced liver tissue damage and improved liver function in an acute intrahepatic cholestasis rat model induced by α-naphthalene isothiocyanate (ANIT) [12]. Swertin extracted from *S. mussotii* alleviated liver injury, inflammation, and cholestasis in rats affected by bile duct ligation-induced liver fibrosis [13].

Iridoid glycosides, the main effective ingredients in the plants of the Gentianaceae family, are cyclopentane-[c]-pyran monoterpenoids with various therapeutic activities, such as neuroprotective, hepatoprotective, anticancer, antidiabetic, antiviral, antiplasmodial, antithrombotic, antitrypanosomal, antioxidant, antihyperlipidemic, and anti-inflammatory properties [14]. The hepatoprotective effects of iridoids and extracts rich in these compounds have been demonstrated in vitro and in vivo [15].

In this study, the enrichment of iridoid glycosides was performed using macroporous resin, and the anticholestatic activity of the total iridoid glycosides from *S. mussotii* (IGSM) was explored in the rat models of ANIT-induced cholestasis. The main composition of IGSM was analyzed using ultra-performance liquid chromatography (UPLC). Then, the effects and the mechanism of the main components of IGSM with FXR were evaluated by molecular docking.

## 2. Results

### 2.1. Effects of IGSM on ANIT-Induced Liver Injury in Rats

**Figure 1:**
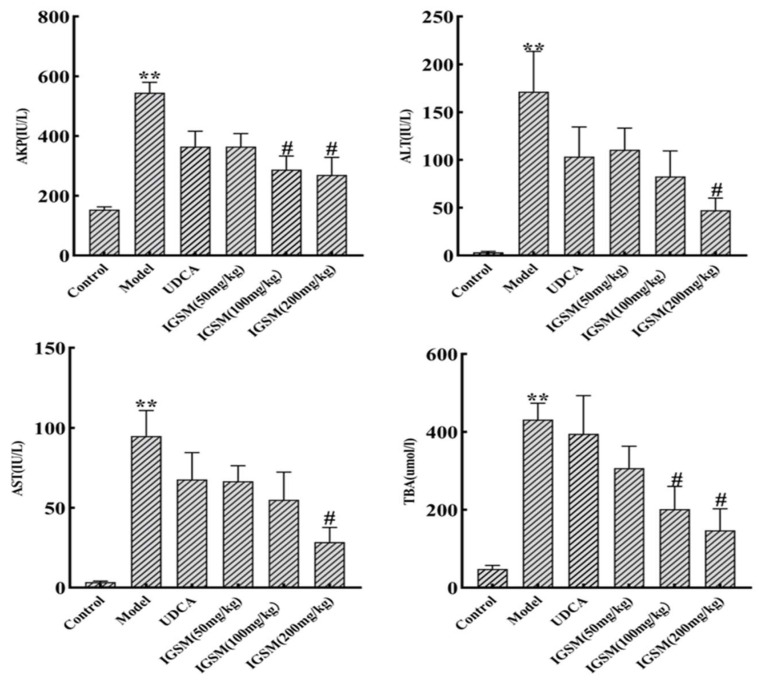
IGSM alleviated liver injury and cholestasis by reducing the serum levels of AKP, ALT, AST, and TBA in ANIT-induced cholestasis rats. ** *p* < 0.01 when compared to the control group; # *p* < 0.05 when compared to the ANIT model group.

The serum AST and ALT levels reflect the degree of liver injury, while the serum levels of TBA and AKP reflect the degrees of cholestasis and bile cell damage, respectively [16]. We observed that the AST, ALT, TBA, and AKP levels in the serum samples of the model group rats were significantly elevated compared with the control rats (*p* < 0.01) (Figure 1), demonstrating the successful establishment of the rat models of liver injury. The administration of a medium dose of IGSM reduced the levels of AKP and TBA in rat serum (*p* < 0.05) and not significantly reduced the levels of AST and ALT. The low-dose group did not significantly reduce the indicators. On the other hand, the serum levels of AST, ALT, TBA, and AKP in the rats in the high-dose IGSM group were markedly lower relative to those of the rats in the model group (*p* < 0.05), suggesting that IGSM alleviated rat liver injury and cholestasis caused by ANIT.

### 2.2. Effects of IGSM on ANIT-Induced Oxidative Stress in Rats

**Figure 2:**
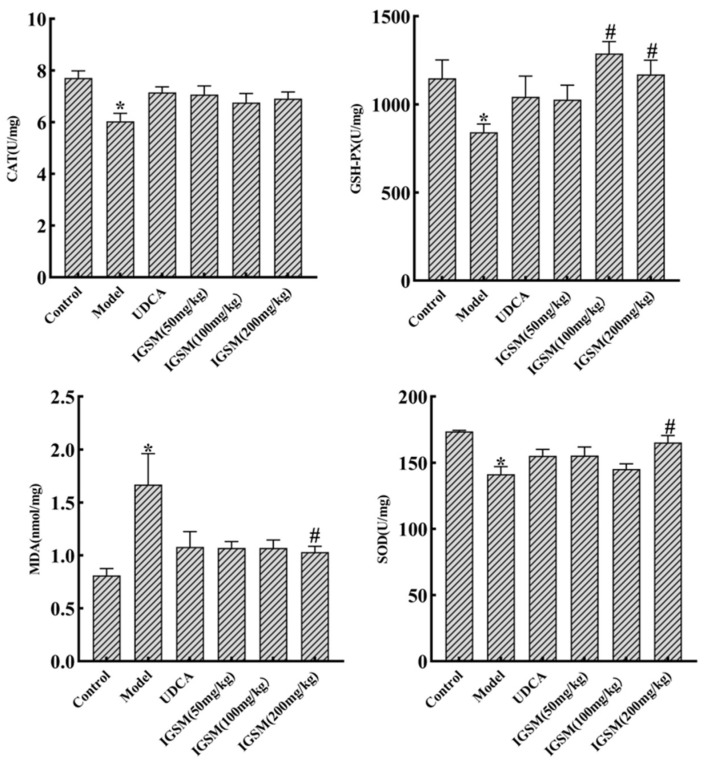
IGSM improved the levels of SOD, MDA, CAT, and GSH-Px in the liver tissues in the ANIT-induced cholestasis rats. * *p* < 0.05 when compared to the control group; # *p* < 0.05 when compared to the ANIT model group.

The MDA levels in the livers of the rats were markedly increased by the ANIT treatment compared to those of the control rats (Figure 2). In contrast, the activities of GSH-Px, CAT, and SOD were evidently reduced by the treatment (Figure 2). These results suggested that ANIT-induced cholestasis increased the level of oxidative stress in rat livers and induced damage to liver tissues. Compared with the model group, the rats in the high-dose group of IGSM treatment displayed a significant decrease in the MDA level and significant increases in the SOD and GSH-Px activities (*p* < 0.05). There was a trend in increasing CAT levels, but it is not significant. However, the medium-dose group only significantly increased the GSH-Px activity (*p* < 0.05) and had no effect on the other indicators. These all indicated that the antioxidant activity of IGSM (200 mg/kg) alleviated ANIT-induced liver injury in rats.

### 2.3. Effects of IGSM on the Morphology of Rat Livers with ANIT-Induced Liver Injury

**Figure 3:**
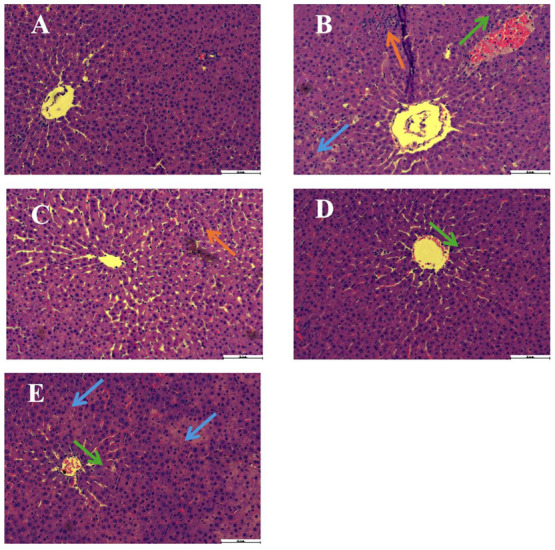
IGSM improved liver pathology in ANIT-induced cholestasis rats (HE stained, 200× magnification). (**A**) Control, (**B**) model, (**C**) high-dose group, (**D**) medium-dose group, and (**E**) low-dose group.

The hepatoprotective effects of IGSM were evaluated by observing HE-stained rat liver tissues. The livers of the rats in the control group showed radial hepatic plates and clear portal areas, hepatic lobules, and central veins (Figure 3), while those of the ANIT-induced rats lost normal liver tissue morphology and showed obvious inflammatory cell infiltration (blue arrow), bleeding (green arrow), and regional necrosis in portal areas (orange arrow). These symptoms were significantly alleviated by the high-dose IGSM treatment.

### 2.4. Effects of IGSM on the mRNA Levels of FXR and Related Proteins

**Figure 4:**
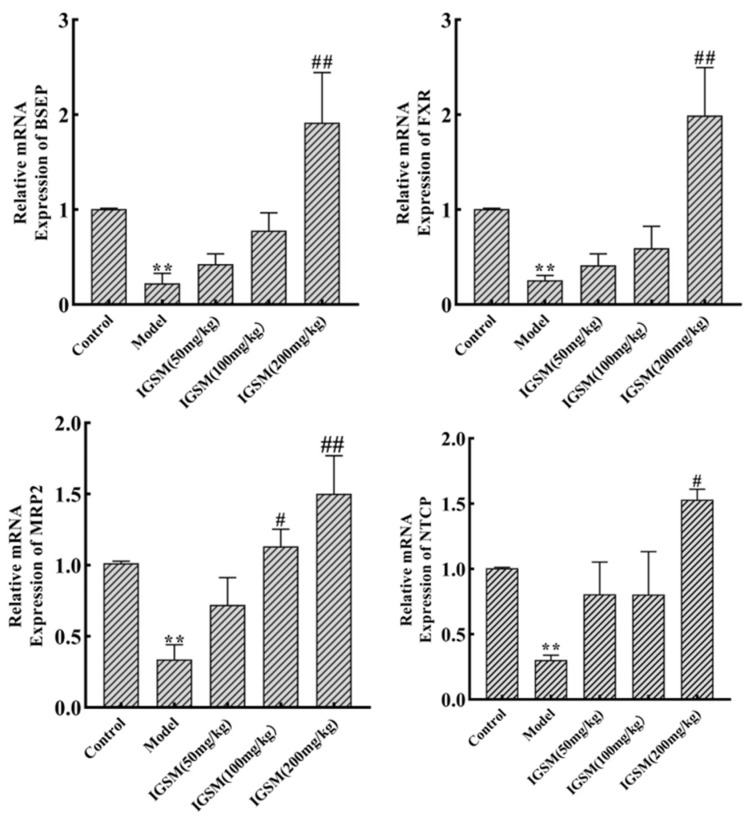
IGSM affected the mRNA levels of FXR and related proteins in the liver tissues of ANIT-induced cholestasis rats. ** *p* < 0.01 when compared to the control group; # *p* < 0.05 and ## *p* < 0.01 when compared to the ANIT model group.

The mRNA levels of FXR and BA transport-related proteins were investigated by RT-qPCR (Figure 4). The mRNA levels of *Fxr*, *Bsep*, *Ntcp*, and *Mrp2* in the liver tissues of the rats in the model group were significantly lower than those in the liver tissues of rats in the control group. The medium-dose group only significantly increased the mRNA levels of *Mrp2* (*p* < 0.05) and had no effect on other indicators. However, on the other hand, the mRNA levels of *Fxr*, *Bsep*, *Ntcp*, and *Mrp2* in the liver tissues of rats in the high-dose IGSM group were significantly higher than those in the liver tissues of rats in the model group (*p* < 0.05). These results indicated that IGSM regulated BA homeostasis by activating the FXR signaling pathway and increasing the levels of BA transporters.

### 2.5. Quantitative Analysis of IGSM

**Figure 5:**
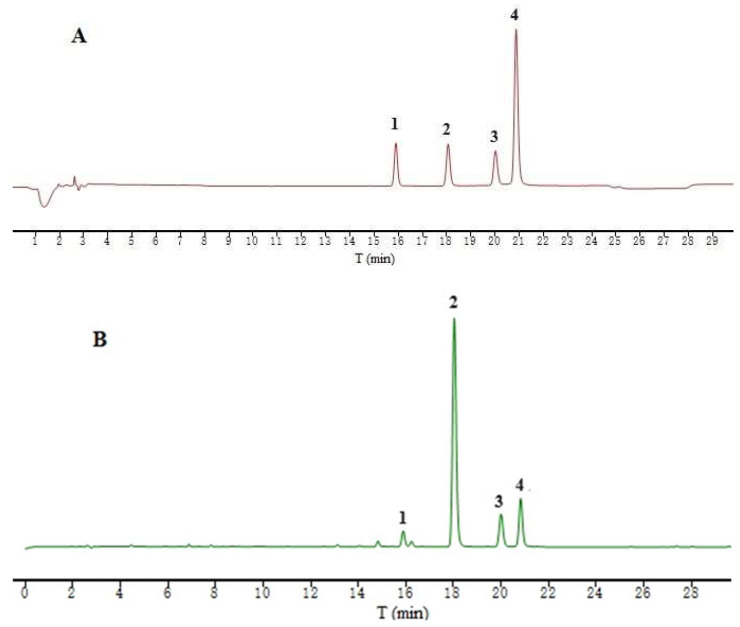
Chromatograms of the four reference substances (**A**) and IGSM (**B**). 1—sweroside, 2—gentiopicroside, 3—swertiamarin, and 4—mangiferin.

Cycloiridoid glycosides are the main active compounds in the Gentianaceae family, among which gentiopicroside, swertiamarin, and swertiamarin are commonly found in the Gentianaceae family [17]. The amounts of swertin, gentiopicroside, swertiamarin, and mangiferin, the four main components of IGSM, were determined at 254 nm by UPLC. The proportion of iridoid compounds in IGSM exceeded 50%, and the gentiopicroside content of IGSM reached 41.39%. This indicated that iridoid glycosides, especially gentiopicroside, were the main active substance basis of IGSM. The regression equation and chromatogram for each standard compound are shown in Table 1 and Figure 5, respectively.

### 2.6. FXR-Activating Effects of the Main Compounds of IGSM

**Figure 6:**
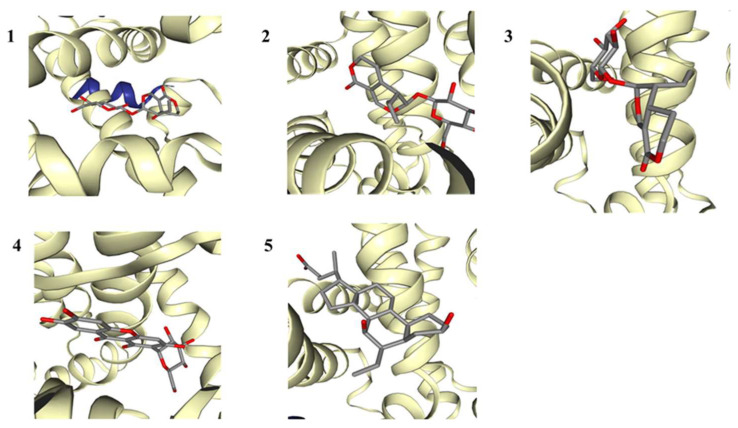
The binding of FXR and compounds: sweroside (**1**), gentiopicroside (**2**), swertiamarin (**3**), mangiferin (**4**), and obeticholic acid (**5**).

**Figure 7:**
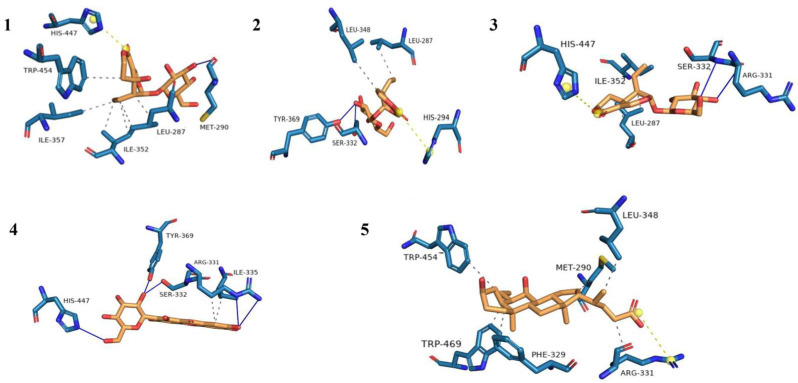
The interactions between FXR and compounds: sweroside (**1**), gentiopicroside (**2**), swertiamarin (**3**), mangiferin (**4**), and obeticholic acid (**5**) by molecular docking analysis.

To understand the interactions between FXR and the main compounds of IGSM, we conducted a molecular docking analysis. The free energies for the docking of the IGSM components with FXR were less than −7 kcal/mol, indicating that the interactions between the components and FXR were stable (Figure 6 and Figure 7, Table 2). Hydrogen bonding and hydrophobic interaction were the main interaction modes (Table 3). Obeticolic acid is an agonist of FXR [18], which was used as the positive control in molecular docking. The free energy for the docking of compound **4** with FXR was lower than that for the docking of the positive control with FXR. It indicated that the binding affinity of this compound with FXR was stronger than that of the positive control.

## 3. Discussion

The rat models of ANIT-induced cholestasis were established as dose- and time-dependent. According to previous studies, elevated ALP levels, elevated ALT levels, chronic inflammation, epithelial necrosis, and focal liver necrosis began 24 h following the oral administration of ANIT at the dose of 75 mg/kg and reached their maximum severity 48 h after the ANIT administration [19,20]. Therefore, this ANIT dose was utilized in this study to induce moderate cholestasis in rats. It has been demonstrated that *S. mussotii* significantly reduced the levels of liver injury indicators such as AST and ALT in mouse serum, alleviating acute liver injury [8,9]. In this study, histological analysis and the measurement of the serum AST, ALT, TBA, and AKP levels were conducted 48 h after ANIT administration, and the results indicated that the ANIT successfully induced cholestatic liver injury. The serum levels of these substances were markedly reduced by the high-dose IGSW treatment compared with those in the model group rats (*p* < 0.05). Moreover, the necrosis, bleeding, and inflammatory cell infiltration in the portal areas of the livers of rats in the high-dose IGSW group were significantly alleviated. Overall, IGSW alleviated liver injury caused by ANIT-induced acute cholestasis.

ANIT induces the death of liver cells and bile duct cells through necrosis and apoptosis. The cell death-inducing activity of ANIT is related to the toxicity of the chemical or the strong inflammation caused by the infiltration of neutrophils into liver cells and the surrounding bile duct. This inflammatory state induces reactive oxygen species generation and oxidative stress, which causes fibrosis, the death of liver cells and bile duct cells, the formation of lipid peroxidation products, and hepatic stellate cell activation [21,22]. Research showed that *S. mussotii* alcohol extract reduced liver damage through anti-lipid oxidation [23]. IGSM significantly reduced MDA levels and enhanced the activities of CAT, GSH-Px, and SOD in rats with ANIT-induced cholestasis in this study. These results suggested that IGSW inhibited cholestasis-induced liver injury through its antioxidant effects.

FXR is activated by BA, and it is considered as the main regulator of BA homeostasis [2]. It has been confirmed that abnormal FXR function triggers disease manifestation in individuals affected by cholestasis [24]. Therefore, activating the FXR signaling pathway is a promising method for treating cholestatic liver diseases. Obeticholic acid is a US Food and Drug Administration-approved drug with the indication of cholestatic liver diseases, and it is known as an FXR agonist [7]. Our results suggested that IGSW was an effective FXR agonist. The molecular docking simulations showed the ability of the main components of IGSW to bind to FXR through hydrogen bonding and hydrophobic interaction. The main active components of IGSM may be potential FXR agonists.

FXR modulates the uptake, synthesis, and excretion of BA by regulating the transcription of genes implicated with BA homeostasis maintenance. The transcription factor can also downregulate BA biosynthesis by affecting the expression of *CYP27A1*, *CYP7A1*, and *NR0B2* genes [25]. On the other hand, FXR positively regulates BA uptake and excretion by affecting the expression of *Bsep*, *Ntcp*, and *Mrp2* genes [26]. BSEP is a hepatocyte canalicular membrane protein, and FXR is the main transcriptional regulatory factor for its expression [27]. FXR promotes the excretion of toxic substances from liver cells into bile by binding to the FXR response elements on the promoter region of the *Mrp2* gene and activating the transcription of the gene [28]. Our results showed that IGSW increased the mRNA levels of *Bsep* and *Mrp2* by increasing the level of *Fxr*. Previous studies have shown that FXR downregulates the expression of the *Ntcp* gene by activating the small heterodimer partner to inhibit the binding of the promoters of the gene to certain nuclear transcription factors [29]. The reduction in the level of NTCP may reduce the uptake of BA and lead to cholestasis or the exacerbation of existing cholestatic liver diseases [30]. IGSW treatment increased the level of *Ntcp* and helped alleviate cholestasis.

In this study, IGSM alleviated ANIT-induced cholestasis and liver injury in rats by decreasing the serum levels of AST, ALT, TBA, and AKP; decreasing the MDA level and increasing the SOD, CAT, and GSH-Px activities in the liver tissues; and increasing the mRNA levels of *Fxr*, *Bsep*, *Mrp2*, and *Ntcp*. This effect was dose-dependent, with significant effects in the high-dose group (200 mg/kg) of IGSM and significant effects in some indicators in the medium-dose group (100 mg/kg), while the low-dose group (50 mg/kg) had no improvement effects.

The main chemical constituents of *S. mussotii* are xanthones, iridoid glycosides, and triterpenes [31]. The extract containing the total iridoids and xanthones from *S. mussotii* promoted bile secretion and reduced the serum levels of AST, ALT, AKP, total bilirubin, and direct bilirubin in the rat models of ANIT-induced cholestatic hepatitis [32]. In this study, swertin, gentiopicroside, swertiamarin, and mangiferin, the four main components of IGSM, were quantitatively analyzed by UPLC as the potential active components of IGSM. Their activity with FXR was confirmed through molecular docking analysis. Gentiopicroside, which is considered as a representative of iridoid glycosides, effectively prevented hepatic BA accumulation and liver injury by upregulating the expression of *NR1H4*, *ABCB11*, *ABCB4*, *CYP7A1*, *ALDH1L1*, and *ABCC4* genes, therefore significantly increasing the hepatic mRNA levels of BA-synthesis enzymes, BA transporters, and ileal BA-circulation mediators [33,34]. Swertiamarin was an effective iridoid glycoside from *S. mussotii* that exerted hepatoprotective effects and mitigated ANIT-induced cholestasis by reversing cholestasis-induced changes in the levels of multiple proteins involved in the FXR signaling pathway [13]. Sweroside attenuated ANIT-induced cholestatic liver injury in mice by restoring bile acid synthesis and transport to their normal levels, as well as suppressing pro-inflammatory responses [35]. The upregulation of FXR mRNA level was observed in sweroside-treated LX-2 cells and in a mouse model of liver fibrosis [36]. In our study, the free energy for the docking of mangiferin with FXR was lower than that for the docking of the other compounds of IGSM with FXR, indicating that mangiferin had a better effect in alleviating cholestasis by activating the FXR signaling pathway. They are the main active compounds in the IGSM for the treatment of cholestasis, which may be potential FXR agonists.

## 4. Materials and Methods

### 4.1. Plant Materials and Reagents

The whole plant of *S. mussotii* was obtained from the Tongtian River Basin (Xiaojin County, Aba Prefecture, Sichuan Province, China) in September 2019, and it was identified by Associate Professor Yubi Zhou from the Northwest Institute of Plateau Biology. A voucher specimen (no. NWIPB-TM-20190901) was deposited in the Key Laboratory of Tibetan Medicine Research, Chinese Academy of Sciences.

Swertin, gentiopicroside, swertiamarin, and mangiferin (all with >98% purity) were purchased from Shanghai Yuanye Biotechnology Co., Ltd. (Shanghai, China). ANIT (purity: >99%) and UDCA were obtained from Sigma-Aldrich Corporation (St. Louis, MO, USA).

The serum levels of AST, ALT, alkaline phosphatase (AKP), and total bile acids (TBAs) and the malondialdehyde (MDA) level, glutathione peroxidase (GSH-Px) activity, and superoxide dismutase (SOD) activity in liver tissues were measured with commercial kits from Nanjing Jiangcheng Bioengineering Institute (Nanjing, China).

*Fxr*, *Besp*, *Mrp2*, sodium taurocholate cotransporting polypeptide (*Ntcp*), and quantitative reverse-transcription polymerase chain reaction (RT-qPCR) primers came from Shanghai Kehua Bio-Engineering Co., Ltd. (Shanghai, China).

HPLC-grade solvents for the UPLC analysis and analytical-grade chemicals were purchased from Merck (Darmstadt, Germany).

### 4.2. Preparation of IGSM

The powder of the whole plant of *S. mussotii* was extracted three times by refluxing it with a 70% aqueous ethanol solution at 65 °C for 2 h each time. The material/liquid ratio was 1:15 (g/mL). The enrichment of iridoid glycosides was performed with DM301 macroporous adsorption resin. Sugars were removed by elution with water followed by elution with a 20% aqueous ethanol solution. The ethanol solution was collected, concentrated, and dried to obtain IGSM. The IGSM yield was 9.42%.

### 4.3. Animals and Treatments

Six- to eight-week-old male Sprague Dawley rats weighing 200 ± 20 g, provided by Jinan Peng Yue Experimental Animal Breeding Co., Ltd. (Jinan, China), were first kept for one week in a controlled environment (22 ± 2 °C) feeding room under a photo period of 12 h light/dark. These animals could intake water and food ad libitum. The experiments on the rats were performed strictly in accordance with the Guide for the Care and Use of Laboratory Animals of Yantai University. The Animal Experimentation Ethics Committee of Yantai University provided approval for the animal experiments (No. YDLL2021R006).

After 1 week of acclimatization, forty rats were randomized to be divided into the following six groups: (1) control (saline, n = 6), (2) model (75 mg/kg ANIT, n = 6), (3) UDCA (75 mg/kg ANIT and 100 mg/kg UDCA, n = 7), (4) low-dose IGSM (75 mg/kg ANIT and 50 mg/kg IGSM, n = 7), (5) medium-dose IGSM (75 mg/kg ANIT and 100 mg/kg IGSM, n = 7), and (6) high-dose IGSM (75 mg/kg ANIT and 200 mg/kg IGSM, n = 7) groups. The above UDCA and IGSM groups were administered by intragastric once a day for 10 consecutive days. On the 8th day, the rats in the control group were injected intraperitoneally with an olive oil solution, while those in the other groups were induced to develop cholestasis by administering 75 mg/kg ANIT in the olive oil solution. The rats were subsequently subjected to a 12 h fasting period 36 h following the drug treatment. The rats were anesthetized by the inhalation of 3.0% isoflurane for 4 min. A glass capillary blood collection tube was used to collect 2.0 mL of blood from the posterior orbital venous plexus of every rat. The rats were sacrificed by cervical dislocation after collecting post-orbital via retro-orbital sinus. Intact liver tissues were carefully harvested from the rats, 2 to 3 pieces of the tissues were fixed in a 4% paraformaldehyde solution for hematoxylin–eosin (HE) staining, a portion of the tissues was used to prepare 10% tissue homogenate solution, and the remaining tissues were stored in enzyme-free cryogenic vials and stored in liquid nitrogen.

### 4.4. Determination of the MDA Level, GSH-Px, CAT, and SOD Activity in Rat Liver Tissues

A portion of the freshly collected tissues was utilized for the preparation of a 10% tissue homogenate in physiological saline. The solution was subjected to a 10 min centrifugation at 3500× *g* to obtain the supernatant; the MDA level, SOD, CAT, or GSH-Px activity were measured using the corresponding commercial assay kits according to the manufacturers’ instructions.

### 4.5. HE Staining of Rat Liver Tissues

The rat liver samples were washed with 0.9% NaCl solution and subjected to paraformaldehyde (4%) fixation, paraffin-embedded, sliced into 5 μm sections, subjected to HE staining, and observed under a microscope (200×) to identify changes in the morphology of the liver cells.

### 4.6. Effects of IGSM on Fxr, Ntcp, Bsep, and Mrp2 mRNA Levels

To determine how IGSM affected the mRNA levels of *Bsep*, *Fxr*, *Ntcp*, and *Mrp2*, we carried out RT-qPCR analysis on the rat liver tissues. The total RNA samples of the liver tissue were extracted for quantification as per the instructions from the Tissue Cellular RNA Rapid Extraction Kit (Shandong Scojet, Qingdao, China). We used a UV spectrophotometer to measure the absorption values at 260 and 280 nm to calculate the purity and concentrations. cDNA was obtained from the reverse transcription of total RNA (1 μL) from each sample according to the SPARKscript||RT Plus Kit (Shandong Scojet, Qingdao, China) (With gDNA Eraser) with an Applied BiosystemsVeriti^TM^ Thermal Cycler. PCR reaction system (20 μL) as follows: 10 μL 2×SYBR qPCR Mix, 1 μL cDNA, 0.4 μL Forward Primer (10 μmol/L), 0.4 μL Forward Primer (10 μmol/L), 0.4 μL ROX, and up to 10 μL RNaseFree H_2_O. The GAPDH gene was amplified separately as an internal control to normalize for gene expression in the samples. The primers used for the amplification of Fxr, Ntcp, Bsep, and Mrp2 genes are shown in Table 4. A StepOnePlus system (Applied Biosystems Inc., Alameda, CA, USA) was employed for the RT-qPCR, the program of which included 40 cycles of 95 °C for 15 s and 60 °C for 60 s. The relative mRNA abundance for each target gene was normalized to that of the GAPDH gene and analyzed with the 2−ΔΔCT method.

### 4.7. UPLC Analysis of IGSM

A methanol solution for IGSM (2.12 mg/mL) was analyzed using an Agilent 1290 Infinity IILC system linked to an Agilent ZORBAX SB-C18 column (250 mm × 4.6 mm, 5 μm). We utilized a formic acid solution (0.1% in water) and methanol, respectively, as mobile phases A and B, for both of which a 0.4 mL/min flow rate was adopted. The following gradient for the mobile phases was set for the assay: the percentage of mobile phase B was increased from 10% to 20% within 5 min, then from 20% to 30% in 17 min, and from 30% to 60% in 13 min. During the entire process of analysis, a column temperature of 35 °C was maintained and the detection wavelength was 254 nm. Additionally, the same experimental settings were applied for the analysis of standards. The standard compounds, swertiamarin, gentiopicroside, swertiamarin, and mangiferin, were precisely weighed at 12.50, 6.00, 5.50, and 5.50 mg, respectively, and brought to a volume of 5.00 mL to obtain solutions of 1.25, 1.20, 1.10, and 1.10 mg/mL. These solutions were then mixed to obtain the mixed solutions of the standard compounds. Based on the peak area of the corresponding compounds in the sample, they were diluted to the corresponding concentrations for the UPLC analysis. The peak retention time in the chromatogram of IGSM was compared with those of the peaks of standard compounds.

### 4.8. Molecular Docking Analysis

Data were analyzed using the GraphPadPrism7.0 software and expressed as means ± standard deviations. We performed one-way ANOVA for comparisons among the experimental groups. Differences were considered statistically significant if *p* < 0.05 or *p* < 0.01.

### 4.9. Statistical Analysis

AutoDock was utilized to simulate the docking between the main components of IGSM with FXR protein. The 3D structure of FXR deposited in Protein Data Bank at https://www.rcsb.org/, (accessed on 29 September 2024) (PDB ID: 3DCT) was downloaded and processed by adding hydrogen atoms and removing all the water molecules [16], while those of the main IGSM components were acquired from PubChem (https://pubchem.ncbi.nlm.nih.gov, accessed on 29 September 2024), added with charges and H atoms, and subjected to free energy minimization for each component. We ensured that the whole molecule of each component was contained in the grid box. The optimal docking model possessing minimal energy was predicted using Autodock Vina, which was considered the most optimal binding structure for an IGSM compound and FXR protein.

## 5. Conclusions

We demonstrated that IGSM alleviated ANIT-induced cholestasis and liver injury in rats by reducing oxidative stress and increasing the mRNA levels of *Fxr*, *Bsep*, *Mrp2*, and *Ntcp*. This study provides data support for the treatment of cholestasis with *S. mussotii*. Iridoid glycosides had a good binding effect with FXR and may be the potential active components of IGSM. IGSM and its active components are expected to be the ingredients of drugs for cholestasis, and future studies may delve more deeply into the FXR regulatory pathway. This study will provide data and guidance for the clinical application of *S mussotii*, further ensuring the application of these results in clinical studies.

## Figures and Tables

**Table 1:** Quantitative analysis results of main compounds of IGSM (n = 3).

NO.	Rt (min)	Compounds	Content in the Sample (%)	Regression Equation	Correlation Coefficient	Linear Range (μg)
1	15.899	Swertiamarin	2.92	y = 2297.3x − 0.1483	1	0.25–0.025
2	18.059	gentiopicroside	41.39	y = 2628.4x + 9.931	1	1.2–0.24
3	20.019	sweroside	6.33	y = 2667.0x + 0.7405	0.9999	0.22–0.022
4	20.872	mangiferin	2.97	y = 11,278x − 14.361	0.9999	0.22–0.022

**Table 2:** Molecular docking information between four main compounds of IGSM and FXR.

Protein Name	PDB ID	Compound Name	PubChem CID	Affinity (kcal/mol)
FXR	3DCT	**1**: Swertiamarin	442,435	−7.8
		**2**: Gentiopicroside	88,708	−7.7
		**3**: Sweroside	161,036	−7.7
		**4**: Mangiferin	5,281,647	−9.0
		**5**: Obeticholic acid	447,715	−8.9

**Table 3:** Molecular interactions between four main compounds of IGSM and FXR.

NO.	Hydrophobic Interactions	Hydrogen Bonds	Other Forces
1	LEU-287, ILE-352, ILE-357, and TRP-454	MET-290	HIS-447
2	LEU-287 and LEU-348	SER-332 and TYR-369	HIS-294
3	LEU-287 and ILE-352	ARG-331 and SER-332	HIS-447
4	ARG-331 and ILE-335	ARG-331, SER-332, TYR-369, and HIS-447	-
5	MET-290, PHE-329, ARG-331, LEU-348, TRP-454, and TRP-469	ARG-331	-

**Table 4:** Primers targeting *Fxr, Ntcp, Bsep,* and *Mrp2* genes for RT-qPCR assays.

Gene		Sequence (5′->3′)
*Ntcp*	Sense	GGACATGAACCTCAGCATCGT
	Antisense	TTGTAGGGCACCTTGTCCTT
*Mrp2*	Sense	GTAGAGAATGAGGCGCCCTG
	Antisense	GGCCGATACCGCACTTGATA
*Bsep*	Sense	TCCTCTGAGCCAAAAGCTGA
	Antisense	CTGCACTCAACAACCCTTTGC
*Fxr*	Sense	CGAGGGCTGCAAAGGTTTCT
	Antisense	TCCCATCTCTCTGCACTTCCT
*GAPDH*	Sense	CCGCATCTTCTTGTGCAGTG
	Antisense	CCGATACGGCCAAATCCGTT

## Data Availability

The original contributions presented in the study are included in the article, further inquiries can be directed to the corresponding author.

## Abbreviations

AKP: alkaline phosphatase; ALT: alanine transaminase; ANIT: α-naphthyl isothiocyanate; AST: aspartate transaminase; BA: bile acid; BSEP: bile salt export pump; CAT: activity catalase; DAPI: 4′,6-diamidino-2-phenylindole; FXR: farnesoid X receptor; GSH-Px: glutathione peroxidase; HE: hematoxylin–eosin; IGSM: the total iridoid glycosides from *Swertia mussotii* Franch.; MDA: malondialdehyde; MRP2: multidrug resistance-associated protein 2; NTCP: sodium taurocholate cotransporting polypeptide; RT-qPCR: quantitative reverse-transcription polymerase chain reaction; SOD: superoxide dismutase; TBA: total bile acid; UDCA: ursodeoxyholic acid.
